# Choosing for others changes dissociable computational mechanisms underpinning risky decision-making

**DOI:** 10.1038/s41598-022-18437-9

**Published:** 2022-08-23

**Authors:** Dominic S. Fareri, Joanne E. Stasiak, Peter Sokol-Hessner

**Affiliations:** 1 Gordon F. Derner School of Psychology, Adelphi University, Blodgett Hall, Rm. 212C, 1 South Avenue, Garden City, NY 11530 USA; 2 Department of Psychological and Brain Sciences, University of California-Santa Barbara, Santa Barbara, CA 93106 USA; 3 Department of Psychology, University of Denver, Denver, CO 80208 USA

**Keywords:** Psychology, Human behaviour, Decision, Social neuroscience

## Abstract

Choices under risk often have consequences for ourselves and others. Yet, it is unclear how the other’s identity (stranger, close friend, etc.) influences risky choices made on their behalf. In a mixed within and between subjects design, two participant groups made three series of risky economic decisions: for themselves, another person, or for both themselves and another person (i.e., shared outcomes). One group made choices involving a same-sex stranger (n = 29), the other made choices involving a same-sex close friend (n = 28). Hierarchical Bayesian estimation of computations underlying risky decision-making revealed that relative to choosing for themselves, people were more risk averse, loss averse, and consistent when choices involved another person. Partner identity was additionally crucial: people became risk neutral and more consistent when choosing for friends relative to strangers. These findings establish that the complexity of the social world is mirrored in its nuanced consequences for our choices.

## Introduction

Imagine that you are a contestant on a game show in which you have the opportunity to make a series of choices between risky options with large potential monetary gains or losses, or safer options that offer no opportunities for monetary gain, but also no loss. When making your choices, you might take into account the subjective value of the risky gain and loss outcomes, how you feel about risk in general, and perhaps even your own history with choices such as this. But what if you were playing this game *for* another person or on a team *with* someone else? Would you simply choose as if you were making the choice for yourself? Or would you evaluate your options differently––to avoid losing money for them, would you become less interested in risk in general, or more careful about balancing gains versus losses, or maybe just more careful and conservative overall? Or would you be riskier for another person to obtain them the largest possible outcome? Would it further matter *who* that person was (i.e., new acquaintance or a close friend)? Multiple factors affect the risks we take—which of them might change when choosing on behalf of another, in which way, and for whom?

While we likely do not regularly appear on game shows, our everyday decisions occur within rich social contexts in which we aim to reduce uncertainty and weigh the (non)social risks and benefits associated with our behavior^1,2^. Indeed, the role of social context in risk-taking has been a topic of intrigue for quite some time^3,4^. Here, we conceptualize risk-taking as a trait that varies *within* individuals in different contexts. Cross-species research suggests that risk-taking increases in social situations. For example, people––particularly adolescents––tend to make riskier driving decisions in the presence of peers, relative to when they are alone, which is paralleled by increases in reward-related neural responses when in the presence of peers^5^. Further, simply observing others’ risk-taking tendencies inspires decision-makers to behave similarly, with money in humans^6^ and addictive substances in rodents^7^. These findings may be explained in part by the desire for social approval^8^, or a derived utility from observing others’ behavior^6^ if we can learn from it^2^.

Yet, the body of work examining the role of social context on risk-taking as a whole is inconsistent at best. One recent meta-analysis reported no differences in economic risk-taking for others relative to the self, but also that people tend to endorse more interpersonal risks (e.g., within dating contexts) for others compared to the self^3^. Another meta-analysis suggested that risk-taking for others in general increases slightly when compared to risk-taking for the self,^4^, but noted that this is highly dependent upon multiple factors including the other’s identity (i.e., patient, child, friend/family) or the frame of the choice (i.e., gain, loss). Often glossed over, however, is the fact that much like other cognitive functions (e.g., memory, attention), risky decision-making is shaped by *multiple component valuation processes,* like feelings about chance, the relative weight of gains and losses, and more (see below). Simply demonstrating a difference in the amount or proportion of risks taken in one context or another does not establish *how* those changes are happening, including when considering risky choices that involve other people. Depending on the specific mechanism(s) affected, we may be able to begin reconciling seemingly contradictory findings^9,10^ and subsequently explain when and why we might expect to find differences in risk-taking in social settings. Furthermore, identifying socially dependent changes in dissociable psychological mechanisms underlying risk-taking can inform targets for cognitive or pharmacological intervention when such choices are maladaptive^11^.

Within the framework of prospect theory^12^, one of the most central theories of decision-making in the past four decades, two key mechanisms are particularly critical when evaluating risky choice options––overall attitudes toward risk (i.e., risk attitudes) and the degree to which we tolerate potential losses relative to potential gains (i.e., loss aversion). People tend to be *risk averse* for gains, favoring smaller but guaranteed positive options relative to larger but uncertain or risky options. People also tend to be *loss averse*, requiring potential rewards to be significantly larger than potential losses^11,12^. While individual differences in risk attitudes are relatively stable, loss aversion has been shown to be malleable as a function of decision context^13,14^. One additional mechanism, choice consistency, has also been examined as part of the stochastic link between value processes and action^14–18^. When choices are entirely consistent, individuals are essentially noiseless or perfectly calculating in their decisions, but as inconsistency rises, participants’ choices become less deterministic.

When making risky choices on behalf of another, it is possible that one or more of the processes underlying our choices, like loss aversion, may shift. For example, we may pursue choice options that are not only of individual interest, but that account for others’ preferences and/or the perceived impact of potential outcomes on others^19–21^. Such a phenomenon may be modulated by the degree of social closeness we feel toward another person^22–25^, which may increase the salience of goals important to the relationship^25^ and help satisfy social needs (i.e., belongingness, experiencing a shared reality)^19,26^. It therefore stands to reason that the component processes underlying risky decision-making may be susceptible to social modulation, which has significant implications for understanding how we make value-based decisions that affect others. While there have been some differences reported across a range of ages in the general pattern of risky choices made for friends relative to others^27,28^ and for parents relative to peers^29,30^, it is unclear from this work what *changes* in decision-making across these different contexts. Thus, we do not yet know the specific ways in social closeness does (or does not) alter the underlying computational processes supporting decision-making under risk.

**Figure 1:**
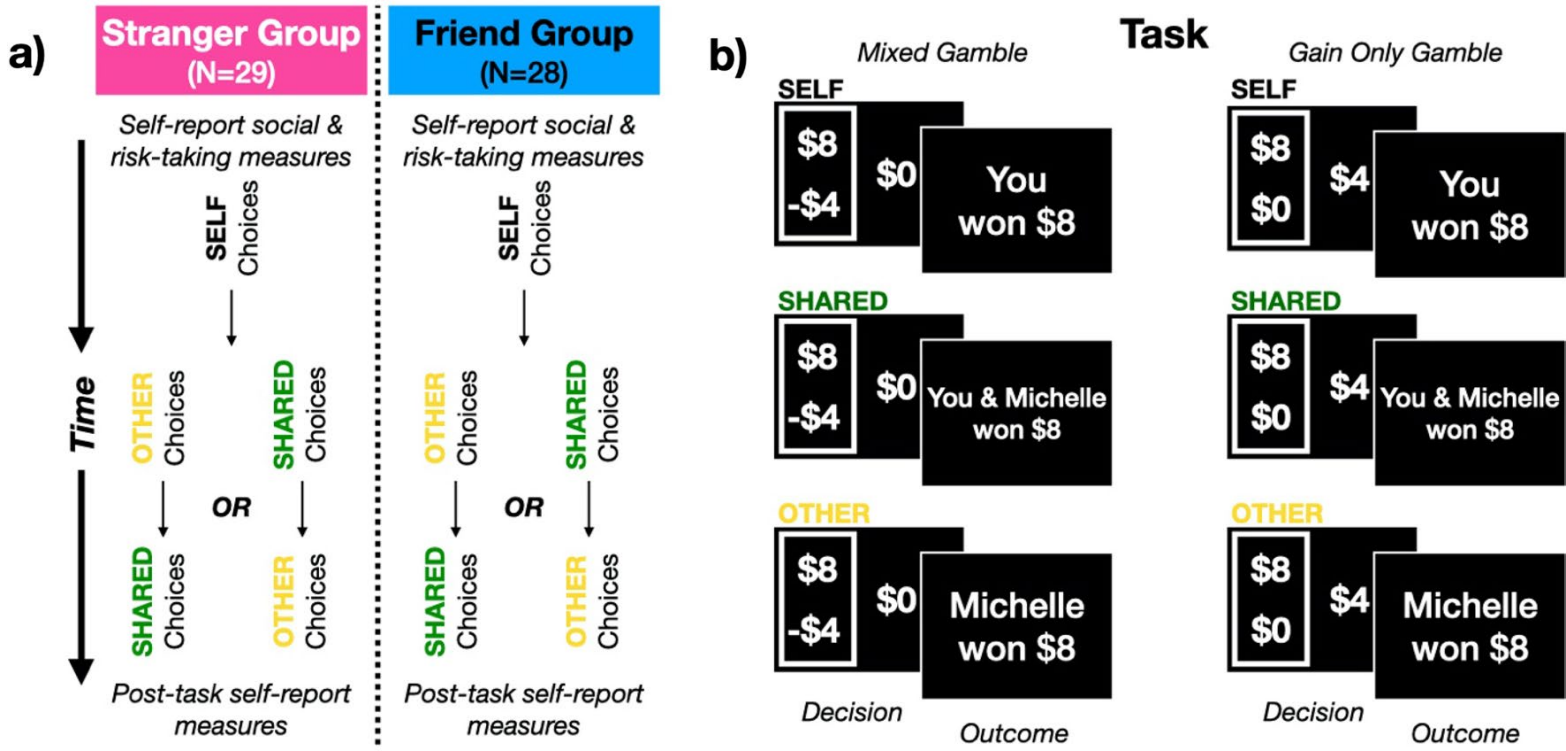
Experimental design and task schematic. (**a**) Participants (decision-makers) were recruited into one of two groups. Decision-makers in the Stranger group (N = 29) were introduced to a sex-matched confederate, while decision-makers in the Friend group (N = 28) were asked to bring a same-sex close friend. Participants were told that the other person (the stranger or their friend) would be taking part in the study with them at different points. At the beginning of the experimental session, decision-makers in both groups completed a series of self-report measures. Following completion of these measures, the experimental task began in which participants chose between risky (50%/50%) monetary gambles or guaranteed monetary outcomes (**b**). Decision-makers in both groups made these choices first in a baseline ‘self’ condition (i.e., outcomes of choices affected only themselves), and then made two other sets of identical choices in a ‘shared’ condition (i.e., outcomes of choices were *shared* between themselves *and* another person) and an ‘other’ condition (i.e., outcomes of choices were *for* another person). The order of these social conditions was counterbalanced across participants in both groups.

The goal of the present study was to characterize whether and how the component processes supporting risky decision-making––risk attitudes, loss aversion, and choice consistency––change when choices are for or shared with another person, and how those changes depend on social closeness. All participants performed a validated risky economic decision-making task^14,15^, altered so the monetary consequences of their choices were sometimes: for themselves only (i.e., baseline non-social context), *for* another person, or *shared* with another person (see Fig. 1).

This within-subjects manipulation uniquely allowed us to assess *changes* in risk attitudes, loss aversion, and choice consistency from how people typically choose for themselves (which is critical because of large baseline individual differences in risky decision-making)^31,32^. We additionally implemented a between-groups manipulation of the identity of the ‘other person’ to be either a same-sex close friend or a same-sex stranger, to allow us to quantify the extent to which any of the changes in decision-making computations in social settings were broadly social in nature, or depended on the presence of a real-life relationship.

Based on evidence of general increases in risk-taking within social (relative to non-social) contexts and an enhanced value placed on rewarding outcomes involving friends relative to others^5,6,24,28,33^, we hypothesized that: (1) people would make riskier choices in social relative to non-social contexts; (2) this pattern would be exacerbated by social closeness, i.e. when choices involved a friend versus a stranger. Given prior work demonstrating malleability of loss aversion, but not risk attitudes^13,14^, we also expected that these changes in risky decision-making would be specifically driven by decreases in loss aversion. We tested these predictions using non-linear hierarchical Bayesian Estimation of a prospect theory-inspired model of risky decision-making.

## Results

### Manipulation checks: self-report measures

We tested whether decision-makers in the friend group reported feeling closer to their partners (friends) than did decision-makers in the stranger group. Based on previous work^24,33^, we expected that greater social closeness would be reported for friends than strangers, and we expected that pairs of friends would report similar views of their friendship. A two-sample t-test revealed that decision-makers in the friend group reported feeling significantly closer to their partners (M = 4.62) than did those in the stranger group (M = 1.93), supporting our hypothesis (*t*_(56.58)_ = 8.36, *p* < 0.001, 95% CI [− 3.33, − 2.04]). Pearson’s correlations also revealed a significant, positive relationship between pairs of individuals in the friend group on social closeness (IOS; r = 0.42, *p* < 0.025) and friendship assessment (PAM; r = 0.8, *p* < 0.001).

We collected responses on individual difference measures of risk-taking, empathy, self-esteem, reward sensitivity and Machiavellianism. Two-sample t-tests on decision-makers from the friend and stranger groups revealed no group differences on: any subscale of the DOSPERT (all *p*’s > 0.2); on behavioral inhibition (*p* > 0.5) or behavioral activation subscales (all *p*’s > 0.3); on self-esteem (*p* > 0.4); on general empathy or related subscales (all *p*’s > 0.4); or on Machiavellianism (*p* > 0.5).

### Post-session ratings

**Figure 2:**
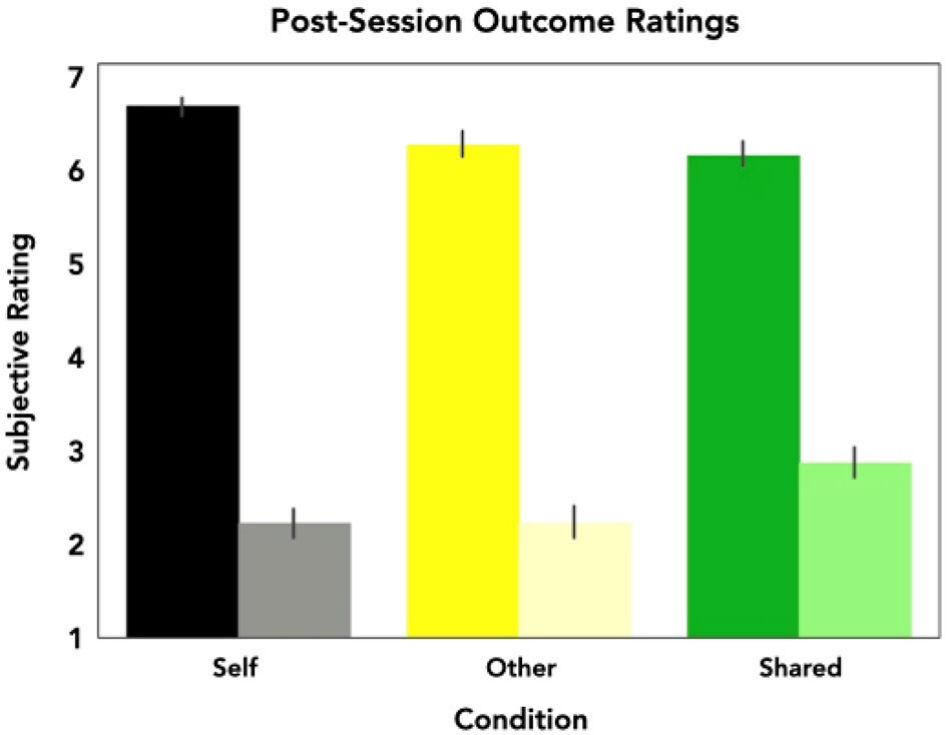
Post-session subjective ratings of experienced outcomes. A 2 × 3 × 2 mixed ANOVA revealed significant main effects of outcome and recipient on participants’ subjective ratings of wins and losses during the task (p’s < .02). Bars represented with saturated colors represent ratings for winning money in each condition; bars represented with pale colors represent ratings for losing money in each condition.

Upon completion of the task, decision-makers in both groups were asked to report how they felt when they experienced wins and losses during the self, other, and shared conditions. A 2 (outcome valence) × 3 (outcome recipient) × 2 (identity) repeated measures ANOVA on decision-makers’ subjective experiences revealed significant main effects of outcome (*F*_(1,55)_ = 451.69, *p* < 0.001, η^2^_p_ = 0.89), and recipient (i.e., self, other, shared) (*F*_(2,110)_ = 4.28, *p* = 0.016, η^2^_p_ = 0.072) (see Fig. 2). Post-hoc analyses further revealed that participants were significantly more excited after a win relative to a loss (*t*_(55)_ = 21.25, *p* < 0.001), and were marginally more excited when they were involved in the outcome as compared to only playing for their partner (shared vs. other: *t*_(110)_ = 2.80, *p* = 0.018; self vs. other: *t*_(110)_ = 2.13, *p* = 0.07, though we note the latter result is only a trend, and so should be interpreted with caution). Furthermore, a significant interaction emerged between outcome valence and recipient (*F*_(2,110)_ = 9.46, *p* < 0.001, η^2^_p_ = 0.153), such that subjects rated winning for themselves more positively than winning for others (*t*_(201)_ = 2.50, p = 0.04) or sharing (*t*_(201)_ = 3.15, p < 0.01), and also rated shared losses more positively than losses incurred for others (*t*_(201)_ = 3.95, p < 0.001) or for the self (*t*_(201)_ = 3.94, p < 0.001). Subjects also reported significantly higher levels of excitement when they won a trial for themselves, relative to losing trials regardless of the receiving agent (all *p*'s < 0.001). However, these effects were not moderated by partner identity (*p* > 0.35).

### Hierarchical Bayesian estimation

We sought to characterize the separable cognitive mechanisms by which social context affected decision computations in two hierarchical models of risky decision-making based on prospect theory^12,34^. Both models estimated the utility of the potential gain and loss outcomes of a gamble as a function of the probability of receiving that outcome and individually estimated parameters for risk attitudes (ρ: values > 1 reflect risk-seeking for gains, and risk-aversion for losses; values < 1 reflect risk aversion for gains and risk-seeking for losses) and loss aversion (λ: values > 1 reflect loss aversion; values < 1 reflect gain-seeking). The likelihood of someone choosing a gamble or the guaranteed option was computed within a softmax function that computed the difference in the utilities of each option while taking into account the consistency of one’s choices (µ). We quantified how participants’ choices changed from baseline by including additive social terms that could change e.g., risk attitudes, loss aversion, or choice consistency on the basis of social indicator variables for ‘other’ (does the involvement of someone else change decisions?); ‘sharing’ (when decisions involve another, is there something different if you share in the outcomes?); and ‘identity’ (does it matter if a friend or stranger is involved?). Full details of our model construction and estimation can be found in the “Methods” section, including how Model 1 and 2 differed, and in the Supplementary Materials S1.

#### Model 1: fixed effects of social factors on decision-making computations

A Bayesian estimation of a hierarchical prospect theory model revealed that at the group level, our sample was risk neutral ($\rho$) (M = 1.07, SD = 0.06, 95% HDI: [0.96, 1.18]), loss averse ($\lambda$) (M = 1.78, SD = 0.20, 95% HDI [1.42, 2.19]), and somewhat consistent (µ) in their choices overall (M = 18.0, SD = 2.14, 95% HDI [13.71, 22.07]) when making choices that only involved themselves.

**Figure 3:**
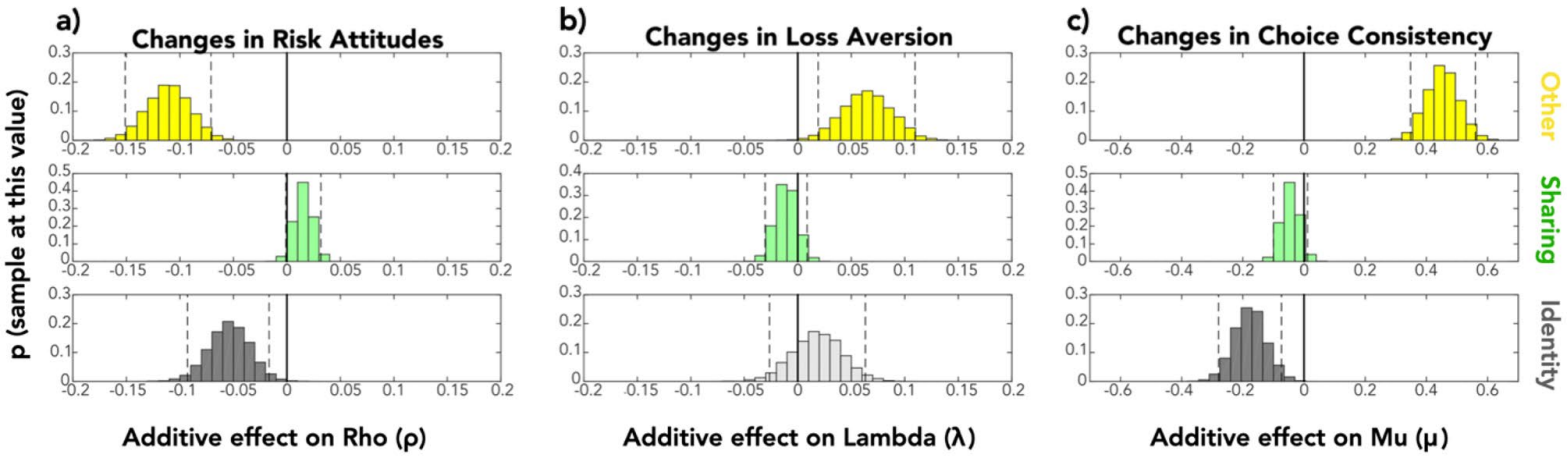
Changes in risky decision computations as a function of social context. Hierarchical Bayesian Estimation of changes in decision-makers’ risk attitudes (**a**), loss aversion (**b**) and choice consistency (**c**) due to social factors revealed consistent additive effects of: (1) ‘other’ on all three computations; and (2) ‘identity’ on risk attitudes and choice consistency. 95% highest density intervals (HDIs) are denoted by dashed vertical lines. No consistent additive effects of ‘sharing’ on any decision computations were observed at the 95% HDI level. Each histogram depicts the distribution of 10,000 samples of that parameter from Model 1. Distributions of samples whose 95% HDI excluded zero are presented in saturated colors, while those for which the 95% HDI included zero are depicted in pale colors.

Social conditions representing whether another person was involved in the choice, whether the outcomes were shared, and partner identity were modeled as fixed effects and indicate additive effects on separable decision processes––risk attitudes, loss aversion, and choice consistency. These analyses revealed reliable additive effects of ‘other’ (a parameter multiplied by a variable coded as + 1 other, 0 self) on all three computational processes––people became more risk averse (M = − 0.11, SD = 0.02, 95% HDI [− 0.15, − 0.07]; Fig. 3a), more loss averse (M = 0.06, SD = 0.02, 95% HDI [0.02, 0.11]; Fig. 3b), and more consistent (M = 0.45, SD = 0.06, 95% HDI [0.35, 0.57]; Fig. 3c) when another person was involved in the decision, compared to when choices affected only oneself. One interpretation of this pattern of results is that people demonstrated an overall tendency toward being more ‘conservative’ or ‘careful’ in choices across the domains of risk, loss/gain, and consistency when choices involved someone else. Because our nonlinear modeling approach used an exponential to implement robust parameter bounds in a manner identical to previous work from our group^15,16^, and similar to extant computational work^18^ (see also “Methods” and Supplementary Materials S1), these additive change terms are not directly in ‘parameter’ (i.e., $\rho$, λ, and µ) space. Their effect size can nevertheless be understood in terms of how they change the mean ‘self’ parameters to their ‘other’ values. In this case, the additive ‘other’ effects shifted the mean $\rho$, λ, and µ values from their respective “self” means of 1.07, 1.78, and 18.0 (see above) to “other” means of 0.96, 1.90, and 28.3 (ignoring effects of sharing or identity, detailed in the following paragraphs and summarized in Table 1).

**Table 1:** Implied mean parameter values for the four social conditions observed in this study.

	Stranger (Identity = + 1)	Friend (Identity = − 1)
Both (shared = − 1)	ρ = 0.89 [0.79, 0.99]	ρ = 1.00 [0.89, 1.11]
	λ = 1.96 [1.55, 2.41]	λ = 1.89 [1.49, 2.33]
	μ = 24.9 [18.7, 31.2]	μ = 35.4 [26.8, 44.7]
Other only (shared = + 1)	ρ = 0.92 [0.82, 1.03]	ρ = 1.03 [0.92 1.14]
	λ = 1.92 [1.50, 2.36]	λ = 1.85 [1.46, 2.27]
	μ = 22.7 [16.9 28.2]	μ = 32.3 [24.4, 40.4]

Values are the implied mean estimates (with 95% HDI values in square brackets) for each of the four conditions defined by our 2 × 2 mixed between/within subjects design. Note that *each cell* contains the effects of Other, Sharing, and Identity, paired with the correct indicator variables and combined with baseline estimates of risk attitudes, loss aversion, and choice consistency. Values of rho (*ρ*) capture risk attitudes; when *ρ* = 1, participants are risk-neutral. Values < 1 indicate risk aversion while values > 1 indicate risk-seeking (for gains; opposite pattern for losses). Values of lambda (λ) capture loss aversion. When *λ* = 1, participants are gain–loss neutral. Values > 1 indicate loss aversion, while those < 1 indicate gain-seeking. Values of mu (*μ*) capture consistency, with higher values indicating greater consistency. Values compare to Self estimates of ρ = 1.07 [0.96 1.18], λ = 1.78 [1.42, 2.19], and μ = 18.0 [13.7, 22.1] (see “Results”).

Estimates of the additive effects of ‘sharing’ on value parameters representing decision processes in the nonlinear model revealed no reliable effect on loss aversion (95% HDI [− 0.03, 0.01]) or choice consistency (95% HDI [− 0.10, 0.01]), and only a weak change in risk attitudes (M = 0.016, SD = 0.008; while the 95% HDI included zero, [− 0.001, 0.03], 96.9% of samples were above zero; note that the coding of the variable by which this parameter was multiplied (see Methods) was + 1 Other-only, − 1 Shared, 0 Self only). This marginal effect would imply average $\rho$ values of 0.96 for choices involving *only* the other versus 0.94 for choices with outcomes *shared* between the participant and the other (ignoring effects of identity, see below), suggesting slightly more risk *aversion* for choices with shared outcomes. The directionality of this effect suggests a specific influence of the context of sharing on risky choice via risk attitudes only such that sharing outcomes with others makes people only marginally more conservative (i.e., risk averse) than when solely choosing for others.

Lastly, reliable effects of ‘identity’ (coded as + 1 stranger, − 1 friend, 0 self, see Methods) on both risk attitudes and choice consistency also emerged in Model 1. Choices involving strangers relative to friends reflected *more* risk aversion (M = − 0.05, SD = 0.02, 95% HDI [− 0.09, − 0.02]) and *less* consistency (M = − 0.18, SD = 0.05, 95% HDI [− 0.28, − 0.07]), but *no changes* in loss aversion (M = 0.02, SD = 0.02, 95% HDI [− 0.03, 0.06]). These effects would imply average $\rho$ values of 1.03 for friends, and 0.92 for strangers, making people risk averse when choosing for strangers, but risk neutral when choosing for friends (assuming choices were made only for the other and not shared; see above). The implied average values of µ (representing choice consistency) were 32.3 for choices involving friends versus 22.7 for choices involving strangers, indicating noisier choices for strangers/more stable choices for friends. Interestingly, loss aversion did not appear to reliably differ as a function of partner identity. For an alternative visualization of the behavioral effects captured by the model, see Fig. S1, which plots the predicted probability of making a risky choice given (1) the difference in expected value between the risky and safe options and (2) the condition in the study, calculated using the group-level average estimates from Model 1.

#### Model 2: individual differences and the relationships between decision computations and social cognitive measures

In Model 2, we estimated our ‘other’ term with hierarchical group-level fixed and subject-level random effects in order to obtain individual estimates of the degree to which participants showed changes in decision processes in social contexts. Doing so allowed us to examine the extent to which any individual differences in risky decision-making for another were related to measures of social cognition and risk perception. See Supplementary Materials S1 for additional context for Model 2’s design.

Model 2 replicated Model1’s findings at the group level. Group-level fixed effects estimates of the effect of the ‘Other’ term on risk attitudes (M = − 0.14, SD = 0.05, 95% HDI [− 0.23, − 0.05]) and loss aversion (M = 0.14, SD = 0.07, 95% HDI [0.02, 0.28]), were consistent with Model 1, finding that choosing when another person is involved increases risk aversion and loss aversion (there were no reliable effects on choice consistency, though the direction of the effect was the same: 95% HDI [− 0.09, 0.37], with 87.8% of samples above zero). Model 2 additionally replicated the pattern of results for ‘Sharing’ found in Model 1: no reliable additive effects of sharing on loss aversion (95% HDI [− 0.03, 0.009]) or choice consistency (95% HDI [− 0.11, 0.006]), and only weak effects on risk attitudes (i.e., slight increase in risk aversion; M = 0.02, SD = 0.009, 95% HDI [− 0.0008, 0.03], 96.7% of samples above zero). Note that post-hoc comparisons of the other effect in Model 2 replicated the pattern of results in Model 1 for strangers versus friends, see Supplementary Material S1.

**Figure 4:**
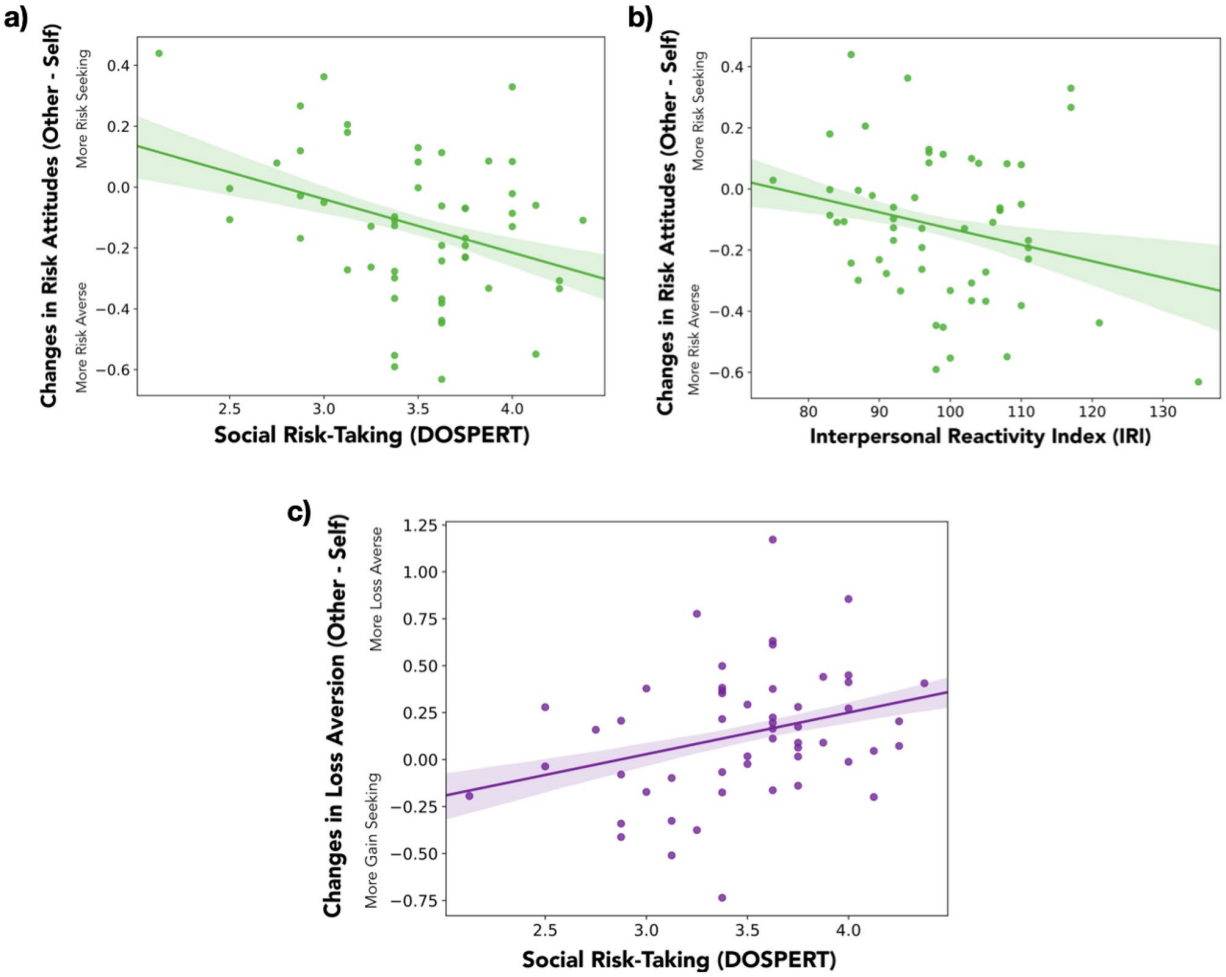
Sensitivity to social context in risky decision-making relates to social cognition and social risk perception. Exploratory analyses revealed associations between subject-level estimates of changes in both risk attitudes and loss aversion (see details for Model 2 in main text) as a function of the ‘other’ term and the self-reported tendency to make socially risky decisions (**a**,**c**; *p*’s < .05) and a composite measure of empathy (**b**; *p* = *.*066).

Critically, obtaining individual estimates of people’s sensitivity to social context during value processing and risk evaluation allowed us to explore relationships between the mechanisms of decision-making and individual difference measures assessing general risk attitudes and empathy. Pearson's correlations (see Fig. 4a,c) revealed significant relationships between the DOSPERT social subscale and risk-related decision processes: participants who reported being more socially risky unexpectedly became more loss averse (*r*_(51)_ = 0.302, *p* = 0.028) and more risk averse (*r*_*(51)*_ = − 0.349, *p* = 0.01) in the social conditions (i.e., when another person was involved in the choice) compared to the self condition, paradoxically suggesting that people who self-report being riskier in social contexts are less risky in multiple ways when deciding for someone else. No significant relationship emerged between the DOSPERT social subscale and the change in choice consistency in social relative to non-social contexts (*r*_*(51)*_ = 0.19, *p* > 0.17). Because taking risks that affect another could involve some degree of consideration of the other’s preferences, we also collected a multidimensional measure of empathy (IRI). A negative trend was also observed in a Pearson's correlation between scores on the IRI and risk aversion in social (vs. non-social) contexts (*r*_*(54)*_ = − 0.247, *p* = 0.066; Fig. 4b), revealing a weak tendency of participants with higher empathy scores to be more risk-averse when making decisions involving another. No significant relationship or trends emerged between empathy and loss aversion (*r*_*(54)*_ = − 0.002, *p* > 0.9) or choice consistency (*r*_*(54)*_ = 0.20, *p* > 0.14) in social contexts. We note that these findings should be interpreted as exploratory and speculative based on the lack of statistical significance in some tests, unexpected patterns in others, and multiple comparisons. However, taken together, these exploratory findings hint that people may compare their attitudes toward risk to those of another person when making choices for others, though we acknowledge the need for replication in future work.

We also examined the degree to which Model 2’s subject-level random effects estimates of the influence of social context on decision processes reproduced the differences identified by Model 1 between choices made for strangers and friends (because Model 2 did not have the “identity” factor integrated into the model). While differences between the average random effects estimates for participants paired with strangers vs. those paired with friends were in the same direction as identified by Model 1, none were significantly different. More specifically, Model 1 identified less risk aversion and more consistency in choices made for friends vs. those made for strangers, but no reliable differences in loss aversion. However, two-sample t-tests of mean subject-level estimates of parameter change for the ‘other’ term from Model 2, between participants in the stranger condition and friend condition, were not significant (all p’s > 0.33). This finding suggests that the strong differences observed in Model 1 were subtle enough in our sample to only be identified when fully leveraging the known structure of the task to maximally pool data across participants while quantifying multiple dissociable component processes underlying risky decision-making.

We conceived of Models 1 and 2 as complementary approaches to fitting the data that capture and emphasize different aspects of the study. While Model 1 emphasized statistical representation of all major design elements in the study, Model 2 emphasized individual differences. Though this perspective renders model comparison of limited value, we performed an exploratory model comparison (which includes a comparison to a model lacking all social factors, Model 0) described in the Supplementary Materials S1.

## Discussion

Our social environments play critical roles in shaping our behavior. This is particularly important within the context of decision-making, considering that many of our choices can have significant consequences (i.e., financial, emotional) for other people. Yet, the specific ways in which the social world changes the component processes underlying risky choices for others is surprisingly understudied. Here, we sought to assess whether and in what way individuals’ unique risky decision-making evaluation and choice processes *change* when those choices affect others. We used hierarchical Bayesian estimation to fit a non-linear model, identifying an overall shift toward conservative behavior, characterized by *multiple changes* in the component processes underlying risk-taking––people demonstrated both increased risk aversion and loss aversion, and were more consistent in their choices when there were consequences for another person. Beyond these overarching social effects, we found more specific patterns characterizing the effects of sharing outcomes with others, and the identity of the person. While sharing outcomes with another person only weakly increased risk aversion, the identity of that person strongly altered decision computations: people displayed *less risk aversion* and *more consistency* when choices involved a real-life friend versus a stranger, possibly reflecting increased certainty about their friends’ preferences (and conversely, relatively more *un*certainty about strangers’ preferences). Taken together, our results support social psychological theories suggesting that closeness may shape our decisions when in the context of others^25^, and hint that participants: (1) may be actively inferring possible preferences for the ‘other’; and (2) that uncertainty in that inference may bias resulting behavior toward conservativism. Most broadly, our approach provides a way to reconcile inconsistencies in the literature on risky decision-making on behalf of others^4^, highlighting the value of breaking decision-making down into its component processes.

Previous work examining risky monetary decision-making in social contexts has identified mixed effects^3,4^, which may result from not dissociating the underlying computations of decision-making. Some studies report similar levels of risk aversion when making choices for the self and for others^9^, and other work indicates that the choice framing (i.e., gain/loss) may influence risk-taking for others^10,35^. Still other evidence points to safer choice behavior (i.e., less money bet on a lottery/higher proportion of safe choices) when choosing for others, particularly friends, relative to the self^27^. However, inferences that can be drawn from these studies are limited because they do not identify which underlying sociocognitive processes drive these coarse behavioral patterns. While a comprehensive review of the subject is beyond the scope of the present article, our approach directly addressed this limitation: we show not only that people are less likely in general to take a risk in social contexts relative to choosing for themselves, but we show *how* such conservative shifts in behavior occur––specifically through reliable shifts toward more risk aversion and lower tolerance of losses.

We expected that the prospect of sharing a potential positive outcome of a risky choice might increase risk-taking via decreases in loss aversion. This hypothesis was based on our previous work showing an increased value placed on rewards experienced in social relative to non-social contexts^24,33^ and evidence of people and rodents engaging in risky behavior together^7,36,37^. To the contrary, we found sharing did not change loss aversion and weakly *increased* risk aversion. Critically, the likelihood of taking a shared financial risk may vary with respect to the perceived similarity in preferences to that person^6^. Thus, the weaker effect of sharing on risky choice in our study may be partially obscured by stronger effects of social closeness to friends relative to strangers.

To this end, we found strikingly consistent additive effects of identity on the computational processes underlying risk evaluation. Choosing for a friend decreased risk aversion (i.e., people became risk neutral) *and* increased choice consistency relative to choosing for a stranger. This pattern of results contrasts with two recent, related psychological studies of which we are aware that investigated risky monetary decision-making involving friends, though we note significant methodological and analytic differences with these other studies. Powers and colleagues^28^ found increased risk aversion when considering choice options involving risky gains or losses for a friend, relative to choices only affecting themselves, suggesting a bias toward protecting close others from potential negative outcomes. Additionally, work by Guassi-Moreira and colleagues^30^ reported no differences in overall levels of risk aversion and trend-level decreases in loss aversion when evaluating risky choices for parents relative to friends. Our decision to include a stranger condition, in conjunction with our unique modeling approach, allowed us to build upon these findings by mechanistically characterizing how the involvement of *specific* close others and strangers in choices *additively shifts people’s idiosyncratic baseline risk attitudes toward risk-taking* as a function of our real-life relationship with that person. More broadly, our findings suggest that we may become more conservative and variable when choosing in social contexts, but to a lesser extent when surrounded by people that we know well.

Our within-subjects manipulation of outcome recipient uniquely enabled examination of how individual sensitivity to the presence of another may relate to individual variability in risk perception and social cognition. Correlations revealed positive relationships between scores on the social subscale of the DOSPERT and both risk aversion and loss aversion. The social subscale of the DOSPERT assesses the degree to which someone engages in socially risky behavior (e.g., openly admitting different opinions than one’s friends), which necessarily involves both mentalizing and valuation processes. We found that the more likely one endorses such behaviors for oneself, the more conservative they are when choices affect others (relative to when choosing for oneself). While trending relationships suggest this pattern could relate to individual differences in empathic abilities, consistent with previous work^9^, we acknowledge these trending results were only found when using a composite self-report measure of empathy, and not specific subscales. Thus, while increased risk aversion when others are involved (relative to choosing for oneself) may depend upon recognizing differences between our and others’ perspectives, this idea must be tested in future work.

Our study is not without limitations. While our task was designed to quantify risk attitudes, loss aversion, and choice consistency, we did not measure other processes that may also be affected by social factors (e.g., ambiguity aversion, probability weighting;^38^), nor did we explicitly assess decision-makers’ ability to gauge friends/strangers risk preferences or examine other processes inherently related to decisions in social contexts (like social perception, affiliation, or theory of mind). While our study focused on dissociating and quantifying decision-making mechanisms that might underlie coarse changes in choices, we hope that future research will be able to more deeply connect the understanding of changes in decision-making processes examined here with other, related processes. We also note our moderate sample size, which was due in large part to challenges recruiting participants in the Friend Group (i.e., coordinating schedules for pairs of people became difficult, resulting in a number of cancellations by participants). As a means to partially mitigate this limitation, we implemented a Hierarchical Bayesian Estimation approach, which leverages each trial as a data point (while the study had 57 participants, it also had > 15,000 trials) and simultaneously constrains estimation of individual-level parameter estimates by the structure of the group-level parameter distributions. Such an approach both allows for more robust estimation and inference than mass univariate analyses, and capitalizes on the growing consensus regarding the importance of repeated measures within participants in terms of maximizing power (Chen et al., 2021). Despite the advantages of this approach to help us detect signal relative to noise in our data, we acknowledge that our ability to generalize to the wider population regarding human social decision-making in general is still in part limited by the number of participants in our study. Future efforts should aim to combine robust within participant power with larger group sample sizes to fully address this concern.

Additionally, our design departed from some of our previous work (e.g., Fareri et al., 2012) which employed within-subjects manipulations of partner. We opted for a mixed design in the present study to reduce the number of conditions experienced (and time required) per individual participant while maximizing our ability to detect within-subject changes in risky choice. However, it is possible that partner identity would have interacted with sharing were this factor also manipulated within subjects. We also note that the friends and strangers were seated in the same room as the decision-makers in the study, even in the baseline condition (albeit at opposite ends of the room). This aspect of our design may have contributed to our baseline group-level estimate of risk attitudes being slightly higher (~ 1) than in previous studies implementing this decision-making task^14,39^. A related point is that participants in this task were always making choices (i.e., taking responsibility) for one specific physically-present other whom they were well aware would potentially be impacted by their behavior (i.e., close friend, stranger introduced atin the laboratory). Thus, our ability to generalize the patterns we observe more broadly to a wider context of decision-making in social contexts may be limited. Lastly, while we counterbalanced the order of the social conditions that participants experienced (i.e., other-only vs. sharing), both social conditions always followed the self-condition in order to clearly establish participants’ baseline decision-making parameter estimates before making choices involving others. This experimental emphasis does present a confound of order effects that we cannot disentangle in this study. We do note, however, that this does not impact any of the estimates related to Sharing or Identity, as these were counterbalanced in order and fully randomized between participants, respectively. In sum, these limitations may be improved upon in future attempts to build upon this work.

There has been interest in understanding differences in risky decision-making in social contexts for a number of years. While recent meta-analyses^3,4^ have suggested inconsistencies in how people take risks in social versus non-social contexts, the underlying literature used to draw this conclusion is arguably fundamentally limited in that it has not assessed different component processes contributing to risky decision-making. Just as it would be incomplete to say that a manipulation does (or does not) change performance on a memory task without addressing whether it affects encoding, storage, or retrieval, studies suggesting that decision-making varies in social contexts should seek to identify the sociocognitive source of that variability. Our nuanced design and analytic approach allowed us to dissociate and quantify specific changes in computational mechanisms underlying risk evaluation as a function of specific, rich, and real-life social manipulations and variables. In doing so we were able to identify distinct behavioral effects in which both the presence of another person and their identity shape the risks we take for ourselves and others.

## Methods

### Participants

Participants were recruited from the student population at Adelphi University via advertisements posted on campus between 2016 and 2019. Participants were recruited into two groups. One group was recruited in pairs, such that upon responding to an ad via email, they were asked to bring a same-sex close friend to the experimental session in which they would take part in an economic decision-making study (Friend Group). A total of 28 pairs of friends were recruited (15F/13 M; mean age = 20.25 years, sd = 1.34). Within each pair of friends, the individual who initially responded to the advertisement will be referred to as the ‘decision-maker’. The other group (Stranger Group, n = 30), upon responding to an ad via email, were asked simply to come to the laboratory to participate in a monetary decision-making study (21F/9 M). One participant was excluded from the Stranger Group because it was later discovered that she had participated in the Friend Group at an earlier date. The final sample of participants in the Stranger Group consisted of 29 participants (20F/9 M; mean age = 20.58, sd = 2.63). Within the Stranger Group, all recruited participants were the decision-makers; the ‘strangers’ were sex-matched laboratory confederates who were portrayed as an additional participant in the experiment and whose identity as laboratory staff was not revealed until the end of the study. To increase statistical power^40^, we included a within-subjects component to our design (see ‘Conditions’ section), collected hundreds of trials per participant and leveraged advanced non-linear hierarchical Bayesian estimation tools that pool data in a structured fashion to maximize signal relative to noise (see ‘Choice Analyses: Bayesian Estimation’ section). Participant recruitment for this study was limited by the need in the friend group to sign up with a close friend, and temporal limits on the internal funding source. All participants provided informed consent, all activities were approved by the Institutional Review Board of Adelphi University. All methods and experimental procedures were performed in accordance with relevant guidelines and regulations governing the approval of this work.

### Procedure

After providing informed consent, participants in both groups first completed basic paper and pencil measures of risk-taking (incentive effects scale)^41^ and perceived interpersonal closeness (inclusion of other in self scale; IOS)^23^. Participants in the friend group (i.e., decision-makers and their close friends), and the stranger group (i.e., decision-makers and confederates) were told to choose the pair of circles, which varied in degree of overlap, that best characterized their relationship in terms of how close they felt to their partners (i.e., close friends and strangers, respectively). Unbeknownst to the decision-makers in the stranger group, confederates did not complete the IOS questionnaire. Participants in the friend group also completed the Personal Acquaintance Measure^42^ as an additional assessment of relationship closeness.

Upon completion of the initial social measures, the decision-maker was seated in front of the experimental computer. The partner in both groups remained seated at a table in the laboratory separated from where the experimental computer was so as to dissociate the effects of choices for others from the potential effects of peer observation^28^. The partner in both groups was instructed that the first part of the task would involve the decision-maker making some choices and that they would be brought into the experiment at the completion of this first component. Once seated at the computer, decision-makers were instructed that they would be performing a task in which they would be making a series of choices about money adapted from our previous work^14^. They were presented with an endowment of $24 which they were told was theirs to use during the task, and that based on their choices, they could potentially add to/lose some of their endowment. Participants were guaranteed $5 for their participation regardless of their task-based performance (see Fig. 1a for a diagram of the study procedure).

#### Gambling task

Decision-makers were instructed that on each trial of the task (see Fig. 1b), they would be making a choice between a 50%/50% monetary gamble and a 100% guaranteed monetary option. Trials featured either mixed gambles or gain only gambles. On mixed gambles, choices were between a risky gamble option offering a 50% chance of gaining some amount or a 50% chance of losing a different amount, and a 100% guaranteed $0 option (i.e., no change to the endowment). On gain only trials, choices were between a risky gamble option offering a 50% chance of gaining money and a 50% chance of $0, and a guaranteed option offering a 100% chance of receiving a smaller gain. We administered a choice set comprised of 90 choices (64 mixed gambles, 36 gain only gambles; the choice set is available on the Open Science Framework at https://osf.io/39b2g/; see supplementary material S1 for additional discussion of the choice set) in each of three conditions (see Conditions, below) for a total of 270 choices. Choices were generated based on the procedure reported by Sokol-Hessner and colleagues^14^. In brief, gamble values for gains on mixed gamble trials were selected from the set of {2, 4, 5, 6, 8, 9, 10, 12}; loss values were computed by multiplying the gain values by factors ranging from − 0.25 to -2. Gamble values on gain only trials ranged from $3.25 to $30. See below (Choice Analyses) for details of the analysis procedure. Within each condition, choices were split into three mini-blocks of 30 trials each, with a brief (< 30 s seconds) break between each.

Decision-makers were presented with choice options for 2 s before a question mark appeared in the middle of the screen indicating that participants had 2 s to indicate whether they wanted to accept the gamble (i.e., take the risk) or reject it in favor of the guaranteed (i.e., safe) option. Participants pressed the number 1 key to accept the gamble, and the number 2 key to reject the gamble. A jittered inter-stimulus interval (ISI) separated the participant’s response from the choice outcome. Decision-makers were presented with text indicating whether they won or lost money if they chose to accept the gamble, or text indicating that they chose the guaranteed option. A short inter-trial interval (ITI) was then presented prior to the next trial. Critically, all participants were informed that each trial was *independent* of all other trials in the experiment––the earnings/losses were not cumulative, but rather choices were to be treated independently, because only one trial would be randomly selected at the end of the experiment, and its outcome realized (relative to their endowment) as their bonus. As such, decision-makers were instructed that they always had $24 to use on each trial, and a loss on one trial did not affect how much money they had on the subsequent trial.

#### Conditions

All decision-makers made choices across three different conditions (Fig. 1b)––self, other, and shared––which were manipulated within-subjects. In order to establish a baseline assessment of risk taking, in both groups decision-makers completed the ‘self’ condition first, in which they made choices whose outcomes would only affect themselves. The order of the remaining two social conditions were counterbalanced across decision-makers. In the ‘other’ condition, decision-makers were told that they were going to complete a similar series of choices except that their choices would instead be made *for* their partner (i.e., close friend in the friend group, stranger in the stranger group). To increase believability, the $24 was given to the partner at the start of this block of trials. In the ‘shared’ condition, decision-makers were instructed that they would again be making a similar series of choices, but that they would be *sharing* the outcomes with their respective partners. Any monetary gains or losses on a given trial were to be split equally with the close friend or stranger. Prior to the start of this condition, the $24 endowment was placed on a desk in the laboratory equidistant from both the decision-maker and their partner.

### Post-task measures and payment

Immediately following completion of the decision-making task, participants answered follow-up questions on 7-point Likert scales to assess subjective experiences of winning and losing in the task in the different conditions (e.g., How excited were you to win money for yourself? For your friend/the other person?). Decision-makers then completed a number of additional self-report measures assessing: general attitudes toward risk-taking (DOSPERT)^43^, empathy (IRI)^44^, self-esteem (Rosenberg self esteem)^45^, general reward sensitivity (BIS/BAS)^46^ and Machiavellian traits^47^. The experimenter then calculated participants’ bonus payment by randomly selecting a single trial from the shared condition of the task. Participants received half of the outcome on that randomly selected trial, as determined by the participant’s choice and the random outcome of the gamble if applicable. In the Friend Group, the remainder of the randomly selected outcome was paid to the decision-maker’s close friend, while in the Stranger condition, participants were informed that the stranger was a member of the laboratory and was not in fact eligible to receive payment.

Following payment, participants were debriefed.

### Behavioral analyses

#### Subjective ratings

Self-reports of social closeness from the Inclusion of Other in Self Scale (IOS)^23^ served as a manipulation check that participants in the friend group felt significantly closer to their friend than did participants in the stranger group to the confederate using an independent samples t-test. We also examined whether pairs in the friend group rated each other similarly on closeness via a Pearson’s correlation. We additionally conducted Pearson’s correlations between friends’ responses on scores on the Personal Acquaintance Measure^42^ as an additional manipulation check to ensure that participants and their close friends had similar conceptions of their relationship. Pearson’s correlations were conducted to test associations between the dyad’s responses on this measure. We note that due to experimenter error, four participants did not complete the DOSPERT and two did not complete the IRI. Post-session subjective ratings regarding decision-makers’ experiences winning and losing money across the different social conditions were assessed via a 2 × 3 × 2 repeated measures ANOVA, with outcome valence (win, loss), condition (self, other, shared) as within-subjects factors, and group (friend, stranger) as a between-subjects factor. All analyses on subjective ratings were two-sided and conducted using Jamovi v1.2^48^.

#### Choice analyses: Bayesian estimation

Our main analyses focused on how social factors change the propensity to engage in risky economic decisions. Note that all models fit to choice data in the current study were hierarchical in nature; that is, they pooled data across participants while retaining the ability to observe individual differences (see below). Compared to non-hierarchical data pooling (ignoring individual differences) or fully-independent analytical approaches (in which each participant’s data are analyzed alone), models with hierarchical structure have been demonstrated to produce more robust group-level inferences, more smoothly accommodate noise (including outlier behavior^18,49,50^), and are often used in examinations of decision-making^15,16,18,51^.

Given that some components of risky decision-making are known to be non-linear, our primary analytic approach consisted of hierarchical Bayesian estimation of a non-linear prospect theory-inspired model of valuation and decision-making under risk (see Supplementary Materials S1 for linear logistic regression analyses of these data). This approach allowed us to directly estimate both group-level and subject-level parameters quantifying the cognitive processes underlying choice, as opposed to classic maximum likelihood methods (including those employed by most linear approaches) that emphasize the identification of simple optimal point estimates for parameters using maximum likelihood estimation^50^.

Hierarchical Bayesian estimation of subject-level and group-level parameters are reciprocally constrained^49^, increasing the stability and reliability of subject-level parameter estimates^50^. The reciprocal constraint uniquely enables hierarchical estimation procedures to fit a model to all trials across all participants simultaneously, in an optimally-weighted fashion that allows differences between participants, while capturing their similarities. This approach is ideal for experimental designs such as this one, that have both many trials per participant and many participants (as opposed to paradigms with very large numbers of trials for only a few participants, or few trials for each of a very large numbers of participants)^18,40,51^. The model applied here has two main components: baseline valuation processes, and the *changes* in those processes due to social factors (e.g. whether one is deciding for a friend or a stranger).

Baseline valuation processes were modeled in a manner similar to that described in prior work^15,16,18^. We first computed the utility associated with the potential gain ($u\left( {x^{ + } } \right))$ and loss $\left( {u\left( {x^{ - } } \right)} \right)$ outcomes of a gamble on a given trial using Eqs. (1) and (2) respectively:

1 $$u\left( {x^{ + } } \right) = p\left( {x^{ + } } \right)*\left( {x^{ + } } \right)^{\rho }$$

2 $$u\left( {x^{ - } } \right) = p\left( {x^{ - } } \right) * \, \lambda *\left( {x^{ - } } \right)^{\rho }$$

where *p(x)* represents the probability of receiving that value, and the parameters *ρ* (rho) and *λ* (lambda) represent risk aversion and loss aversion, respectively. The probability of choosing a gamble was estimated by taking the difference between the utilities of the gamble and guaranteed options on a given trial using a softmax function (Eq. 3):

3 $$p\left( x \right) = \frac{1}{{1 + e^{{\left( { - \mu *\left( {u\left( {gamble} \right) - u \left( {guaranteed} \right)} \right)} \right)}} }}$$

in which the parameter *µ* represents participants’ choice consistency.

The parameters in Eqs. (2) and (3) (*ρ*, *λ*, and *µ*) all influence the decision to accept or reject a risky choice option, but for different reasons—this model allowed us to dissociate and quantify those effects. Rho (*ρ*) reflects risk attitudes, regardless of whether choices involved the potential for a gain and loss (i.e., mixed gambles) or only gains. A value of *ρ* = 1 reflects a risk neutral individual; *ρ* > 1 indicates a pattern of risk seeking for gains and risk aversion for losses; *ρ* < 1 reflects a pattern of risk aversion for gains and risk seeking for losses. Lambda (*λ*) reflects loss aversion, which is defined as the weight on potential losses relative to potential gains. When *λ* = 1, individuals weight losses and gains equivalently (i.e., gain–loss neutral); *λ* > 1 indicates loss aversion, such that losses carry a greater weight per dollar than gains, and *λ* < 1 indicates gain seeking, such that gains outweigh losses of the same magnitude. Mu (*µ*) captures choice consistency, reflecting a given participant’s internal consistency (i.e., stability) in their choice behavior: greater values of *µ* reflect greater consistency in choice behavior.

We estimated not only individual-level *ρ, λ*, and *μ* values, but group-level mean and variance parameters for each *of ρ, λ*, and *μ*, around which the individual-level estimates were distributed. This hierarchical approach therefore both represented each original, raw datapoint (i.e. each choice, instead of relying on individual-level summary statistics) and took advantage of the known structure in the data by estimating parameters simultaneously at both the participant and the group level. This maximizes the signal-to-noise ratio by smoothly reducing the influence of outliers and noise, with the consequence that ‘sample size’ is most accurately understood not simply in terms of number of participants (57), but the total number of *datapoints* (15,390 choices; 270 choices for each of 57 participants), as the hierarchical model-fitting process effectively and intelligently leverages each of those datapoints when estimating fit. Estimates derived from, for example, the same number of participants with only 20 choices per person (1140 choices; 20 choices for each of 57 participants) would reflect an order of magnitude less data, and would thus be considered much less robust, ceteris paribus, despite a nominally identical (participant) sample size^40^. This approach to appreciating the contributions of both sample size and number of trials per participant to statistical power is conceptually identical to that in other highly repeated measures environments (i.e., task-based neuroimaging^40^).

While the above parameters captured participants’ overall attitudes toward risk, loss aversion, and choice consistency, we wanted to additionally quantify the effects of social factors of interest––other, sharing, identity on estimates of *ρ*, *λ*, and *µ*. That is, how the presence of other people (and *which* other people) affected risk attitudes, loss aversion, and choice consistency. We modeled these social variables as indicators as follows. First, we modeled the effect of *other*––does the involvement of someone else change decisions using the self as the baseline to which the presence of another was compared (self (0), another person (+ 1)). We then constructed *sharing* and *identity* contrast-style variables, meant to be additive on top of the effect of *other*, that in essence asked whether decisions for another person were additionally affected by other details of the social situation. The *sharing* variable examined whether decisions involving others were different if participants did or did not share in the outcomes (self (0), for another person only (+ 1), or shared between self and other (-1)). Finally, the *identity* variable assessed whether it mattered who was involved (self (0), stranger (+ 1), friend (− 1)). We captured these effects with additive terms in two different models: Model 1 and Model 2.

#### Model 1: Fixed effects of social factors on decision-making computations

In Model 1, each social factor of interest (other, sharing, identity) had its own term for affecting each choice parameter ($\rho$, λ, and μ). This resulted in a total of 9 additive social terms (3 social factors for each of the 3 main prospect theory parameters) which could increase or decrease the relevant group-level choice parameter. These social factors were implemented as fixed (group-level only) effects.

The manner in which each additive social factor of interest influenced the baseline valuation processes was the same in each case. For example, risk aversion was affected by three additive social factor parameters of $\delta \rho^{other}$, $\delta \rho^{sharing}$, and $\delta \rho^{identity}$. A given participant’s risk aversion on a given trial was then calculated as the sum of their baseline risk aversion and these three additive $\delta \rho$ parameters multiplied by the unitary (− 1/0/ + 1, as applicable) other, sharing, and identity vectors described above. For example, in equation form, risk aversion for participant “s” on trial “t” was calculated as follows:

4 $$\rho_{st} = \rho_{s} + {\text{other}}_{t} *\delta \rho^{other} + {\text{ sharing}}_{t} *\delta \rho^{sharing} + {\text{ identity}}_{t} *\delta \rho^{identity} .$$

Our model was estimated using a Markov Chain Monte Carlo (MCMC) sampling approach in rStan^52^ across four chains for a total of 10,000 samples/parameter. Effects of social factors on parameters of interest were evaluated with 95% highest density intervals (HDIs). The HDI identifies the interval such that every point inside has higher credibility than any point outside the interval. If a 95% HDI excludes zero, that would indicate that given the observed data, the most credible values of that parameter were non-zero. See Supplementary materials S1 (‘Hierarchical Bayesian Model Configuration and Estimation Details’ section) for additional details about model configuration including the implementation of parameter boundaries and model estimation.

#### Model 2: individual differences and the relationships between decision computations and social cognitive measures

We implemented Model 2 with two goals in mind. First, we aimed to identify how individual differences in the effect of social factors on baseline value parameters related to individual difference measures obtained via self-report (see below). To accomplish this, Model 2 was identical to Model 1 with respect to baseline value parameters ($\rho$, λ, and μ), but the additive terms of Other on each of ⍴, λ, and μ were modeled as *mixed* effects (i.e., including a group-level mean and standard deviation around which individuals’ ‘Other’ effects were distributed). Second, we sought to identify the extent to which stranger/friend differences might appear in the Other term (which was being allowed to vary by participant) without assumptions that such differences existed at a between-subjects level. Thus, Identity was not explicitly modeled as a factor in Model 2. The effects of Sharing on each of ⍴, λ, and μ were modeled as fixed effects (as in Model 1) for simplicity. See supplementary materials S1 for more information.

We also wanted to assess the relationship between choice behavior and traits that may underlie a propensity for (or aversion to) risk-taking in social contexts. Individual participants’ parameter estimates were examined in relation to scores of empathy (IRI) and risk attitudes (DOSPERT) through two-sided correlational analyses conducted in Jamovi. We were primarily interested in whether and how such individual differences may relate to behavioral shifts that take place in the presence of others.

Though we conceived of Model 1 and Model 2 as complementary approaches to probe different questions with respect to our data, we did perform an exploratory model comparison. To evaluate model fit and compare the fit between models, we calculated the widely applicable information criterion (WAIC) using the log-likelihood calculated for each data point for each sample. The WAIC was calculated and compared using the ‘loo’ package in R. See Supplementary Materials S1 for more information.

## Supplementary Information

Supplementary Information.

## Data Availability

The dataset generated and analysed during the current study are available on the Open Science Foundation at https://osf.io/39b2g/.
