# Supplementary material for: Choosing for others changes dissociable computational mechanisms underpinning risky decision-making
Source: Sci Rep. 2022 Aug 23;12:14361. doi: 10.1038/s41598-022-18437-9 (PMC9399086; doi:10.1038/s41598-022-18437-9)
Supplement: Supplementary file 1 — Supplementary Information. [file 41598_2022_18437_MOESM1_ESM.docx]

**SUPPLEMENTARY MATERIALS, METHODS & RESULTS**

**Materials**

**Choice Set**. The choice set used in the current study featured two trial types: mixed trials, and gain-only trials. Mixed trials (also called gain-loss trials) are defined by the risky option having possible outcomes that are positive and negative, each received with 50% probability if that option is selected. The guaranteed or certain alternative is $0 on every trial. Gain-only trial types are defined by the risky option having positive or zero possible outcomes, received with 50% probability if that option is selected, and a guaranteed or certain alternative of a (smaller) positive amount.

These two trial types were included to allow the dissociation of the two distinct processes of risk attitudes and loss aversion. Both risk attitudes and loss aversion affect decision-making on mixed trial types, though for different reasons. For example, risk attitudes can increase risky gain-loss option rejection because of diminishing sensitivity/a distaste for uncertainty. Loss aversion can *also* increase risky gain-loss option rejection because the possible loss outcome subjectively outweighs the possible gain outcome. In both cases, the gross behavior (rejection of a risky gain-loss option) is the same, making these two choice processes impossible to truly disentangle with only mixed trial types. The inclusion here of gain-only trial types allows the dissociation of risk attitudes and loss aversion, because loss aversion does not play a role on gain-only trials as no losses are present – only risk. This issue and its solution has been discussed elsewhere in detail (1–3).

A third possible trial type, loss-only trials, is sometimes included in risky decision-making tasks (4,5). On loss-only trials, participants choose between a risky option with possible outcomes that are negative or zero, and a guaranteed or certain option that is a smaller negative amount. We elected not to include those trials here for two main reasons: 1) the loss-only trial-type is strictly aversive, and so can reduce engagement or lead participants to engage in globally rational, if experimentally-problematic, strategies like not responding at all, and 2) the main additional capability that such trials would lend is the ability to disentangle or separately estimate the utility function curvature in the gain and loss domains, despite a lack of consistent evidence that such curvatures do systematically differ within and across participants (see page 661, Booij & van de Kuilen, 2009). Thus, given the small and limited additional benefit in the current situation, potential experimental problems introduced, and simple constraints on the length of the decision-making task (made more challenging by the use of three conditions within-participant), we opted to not include loss-only trials in the current study.

**Methods**

**Choice analyses: Logistic Linear Regression***.* Our first approach aimed to characterize the likelihood of accepting or rejecting a gamble as a function of both who the outcomes would affect (i.e., self, other, shared with another) and the identity of that other person (i.e., friend, stranger). We conducted a two-sided mixed effects logistic linear regression in R statistical language using lme4 (v1.1.23) and lmerTest (v3.1.2) (7,8). Choice behavior (risky (1), safe (0)) represented the outcome variable and was regressed on three social factors: *other*––does the involvement of someone else change decisions? (self (0), another person (+1)); *sharing*––when decisions involve another, is there something different if you share in the outcomes? (self (0), for another person (+1), or shared between self and other (-1)); and *identity*––does it matter who is involved? (self (0), stranger (+1), friend (-1)). Based on results from previous work from our group (9,10), we included a sharing*identity interaction term to examine whether sharing with a friend or stranger differentially predicted choice behavior. We also included regressors representing all monetary amounts on a given trial (i.e., the amount of possible gain, possible loss and guaranteed option) in our model to ensure that any emerging effects of social factors on risky choice were over and above any effects of the potential monetary consequences associated with a given choice. We included fixed effects for all regressors in our model and additionally estimated randomly varying slopes for the monetary values present in a given choice as a function of subject.

**Hierarchical Bayesian Model Configuration and Estimation Details**. Directly following procedures used elsewhere (11–13), to facilitate the use of MCMC sampling methods, the model was configured so as to implement conservative parameter bounds (e.g. that $⍴$, λ, and μ are all strictly positive) using mathematical transformations on unbounded variables. Sampling could then proceed in unbounded space, while still using such boundaries. For each of $⍴$, λ, and μ, the parameter was sampled in unconstrained space (“sampling space”), from negative to positive infinity, but those values were then passed through an exponential function prior to application to dollar amounts within the model (“application space”) as in Equation S1, below.

Eq. S1 $\theta= e^{\beta}$

$\theta$ here represents $⍴$, λ, and/or μ (application space), while β represents the variable over which sampling actually occurred (sampling space). While β is unconstrained, by raising *e* to the β, the resulting $\theta$ is strictly positive. Values of $⍴$, λ, and μ in the main text are reported exclusively in application space (i.e. $\theta$), after the transformation was applied.

Equation 4 in the main text, detailing how Other, Sharing, and Identity additive factors were included was similarly implemented within the exponential to prevent overflow errors with summation that would violate a bound (in this case, crossing zero to negative values of the parameter). Thus the implementation of Equation 4 within the MCMC sampling framework for participant ‘s’ on trial ‘t’ was as in Eq S2, below.

Eq. S2 $\theta_{s,t} = e^{(\beta_{s} + {other}_{t}*\delta\beta^{other} +{sharing}_{t}*\delta\beta^{sharing} + {identity}_{t}*\delta\beta^{identity})}$

Because the *δ* terms are only interpretable as *changes to* baseline parameter values (β_s_), *δ* term values are reported in the main text in untransformed *sampling space*, as they do not have a corresponding standalone ‘application space’. In other words, they are never present without the baseline parameter values.

Note that the above procedures for configuring and implementing the model, and these basic bounds, are both standard in the field in general (e.g. that the loss aversion parameter is strictly positive) and are exactly as has been done in our prior research (14).

*δ* terms in Model 1 (featuring effects of Other, Sharing, and Identity on each of $⍴$, λ, and μ) were all modeled as ‘fixed effects’, meaning that each was implemented as a single parameter shared across all participants.

Model 2, fit for the purposes of identifying between-subjects variation related to other social cognitive measures of interest in the study, fit the effect of Other as a mixed effect on each of $⍴$, λ, and μ, directly estimating a group-level mean and standard deviation, around which individuals’ effects of Other were distributed. The additional effect of Sharing was modeled as a fixed effect, as in Model 1, on each of $⍴$, λ, and μ.

Identity was not included in Model 2 for two main reasons. First, because the *intent* behind Model 2 was to quantify individual differences in decision-making, and examine the extent to which those differences separated on the basis of partner Identity (i.e. stranger vs. friend), without assuming such differences. We examined potential separation on the basis of partner identity by calculating sample-wise differences between the mean self-other parameters for those participants paired with friends versus those paired with strangers, and examined the 95% credible interval of those sample-wise differences in means (see below). The second reason we did not include Identity in Model 2 was that the use of mixed effects for Other effectively precludes doing so. Because every participant had a unique value for Other, a group-level fixed-effects term for Identity could be somewhat co-identified with the individual estimates of Other. Put another way, if the participants’ different Other estimates sorted so that all participants in the Stranger condition were +1 for Other, and those in the Friend condition were -1, it would be *perfectly* collinear with a fixed-effects Identity term).

We see Models 1 and 2 as complementary. Model 1 is best thought of as a conservative test, leveraging most of what we as experimenters know about the structure of the task (but assuming that people are very similar to each other in terms of the effects of social factors on decision-making. However, we note that Model 1 still accounts for individual differences with mixed effects modeling of baseline decision-making parameters for risk attitudes (rho), loss aversion (lambda), and choice consistency (mu). Model 2 on the other hand, includes more sophisticated modeling of the individual differences in the effects of social factors, though it does not leverage everything we know structurally as experimenters.

**Priors and Distributions.** In Models 1 and 2, the baseline $⍴$, λ, and μ parameters were modeled in sampling space (see above) as follows: a mean (with a normal (0,30) prior) and standard deviation term (cauchy (0,2.5) prior), defining a group-level gaussian or normal distribution around which individuals’ baseline $⍴$, λ, and μ parameters were distributed.

In Model 1, additive Other, Sharing, and Identity fixed effects parameters for each of $⍴$, λ, and μ all had normal (0,30) priors. In Model 2, individuals’ additive Other terms for each of $⍴$, λ, and μ were modeled as follows. There was a group-level mean (with a normal (0,30) prior), and standard deviation (with a cauchy (0,2.5) prior), defining a group-level gaussian or normal distribution around which individuals’ Other terms were distributed. Sharing terms were modeled as fixed effects as in Model 1 (with a normal (0,30) prior).

The sampling procedure used with rstan included a long burn-in period which greatly reduces the impact of priors on estimates of the posterior by discarding the first 2,500 samples in each chain (of which there were 4), and only retaining and analyzing the final 2,500 samples in each chain. Combined with the uninformative (broad) priors, such a sampling procedure results in a posterior that almost entirely reflects the likelihood (not the prior).

**Hierarchical vs. non-hierarchical approaches to data analysis**. In the main text, we referred the additional power that hierarchical approaches bring to data analysis. In brief, by smartly pooling data, one gains the ability to leverage all known data simultaneously, without discarding known differences in the sources of data (e.g., from participant #1, participant #2). Such an approach allows one to specify which data-generating processes are thought to be constant or shared, similar, or truly distinct, and how. Pooling data completely necessarily ignores information about, for example, participants being different from one another, while analyzing participants completely independently of each other amounts to a strong assumption that there is *nothing* one can learn about a given participant from other participants. Scientifically speaking, the antithesis of this latter assumption is indeed widely held – that we absolutely *can* learn about a person or group of people from other peoples’ behavior, feelings, responses, etc).

There are at least two main downsides or risks with hierarchical analytic techniques: first, such techniques often require a degree of technical sophistication to appropriately specify hierarchical statistical structures, and they can be difficult to estimate or troubleshoot, especially if these structures are complex. This can make hierarchical models hard to use, especially with novel participant types or groups, experiment structures, and/or models. The second risk is that with the specification of structure comes the concern that one might misrepresent the structure, or ‘get it wrong’ (though arguably this kind of risk is inherent any time one attempts anything at all). Occam’s Razor thus still applies (that simpler models, structures, or approaches should be preferred), and ideally structures should be empirically or theoretically motivated (or possibly both) prior to their use.

Thus, non-hierarchical approaches are especially useful in a limited set of scenarios: when the structure of the data is very complex and unknown, when no significant individual differences are expected (warranting non-hierarchical data pooling).

Others have written extensively on this subject, including Nilsson et al, 2011, Daw 2009, Wiecki 2013, Scheibehenne & Pachur 2014, Fleming 2017, Guest & Martin 2021, among others.

**Results**

**Linear Estimation of the Likelihood of Gamble Acceptance.** Our first question concerned whether the likelihood of accepting a gamble (vs. a guaranteed outcome) on a given trial differed as a function of the surrounding social context, within a simple linear framework. A preliminary mixed effects logistic linear regression first revealed the expected simple effects of the values of the options under consideration (b_risky gain_ = 0.34, SE_risky gain_ = .05, z_risky gain_ = 7.56; b_risky loss_ = 0.84, SE_risky loss_ = .07, z_risky loss_ = 11.79; b_safe alternative_ = -0.70, SE_safe alternative_ = .08, z_safe alternative_ = -8.57; all *p*’s < 0.001). The model also identified that participants were significantly less likely to gamble when another person was involved in the choice (b_other_ = -0.49, SE = 0.05, z = -9.76, *p*<0.001). This effect was exacerbated when choices were *shared* with another person (b_sharing_ = 0.08, SE = 0.03, z = 2.77, *p* = 0.006). However, no effect of partner identity (friend vs. stranger) was observed (*p* > 0.9) nor was there a significant interaction of sharing and identity (*p* > 0.3) when considering the overall likelihood of accepting a gamble.

While this approach established that the social contexts manipulated in the current study had effects on decision-making, it was limited in two ways that reduce the degree to which we can draw strong inferences from these findings. First, these effects were measured in a linear framework (even though some components of risky decision-making are known to be non-linear (15)). Second, this approach modeled a direct effect of social factors on risk-taking, and did not model the component processes of decision-making or the influence of our social contextual variables on those component processes. Our hierarchical Bayesian estimation of nonlinear models of valuation and decision-making described in the main text addresses the limitation of linear models in this context.

**Differences in Model 2 Between Participants Paired with Friends Versus Those Paired with Strangers.** The intuition behind this analysis was to examine the subject-level estimates of the effect of Other for possible differences between those participants who were paired with friends and those paired with strangers, as Identity (Friend, Stranger) was not explicitly included in Model 2. In other words, to what extent do effects of partner Identity arise out of the model, even when they are not included?

On each sample of Model 2 (10,000 in all), we calculated two simple averages: the average Other effect for those participants paired with Friends, and the average Other effect for those participants paired with Strangers. Note that the simple average discards participant-level uncertainty in these parameter estimates, with the result that they should be interpreted with caution. We then examined the sample-wise *Friend - Stranger* difference in those averages across all 10,000 samples. Results were broadly similar in direction to the findings of Model 1 (which found less risk aversion and more consistency for Friends as compared to strangers, but no meaningful change in loss aversion), though we note the effects were weaker overall (i.e., all 95% credible intervals included zero), likely because the model assumed no differences between these groups in the first place.

The average Friend - Stranger difference in risk attitudes was 0.035 (reflecting a higher risk attitudes parameter, which means less risk aversion/more risk-seeking for gains), with a 95% highest density interval of [-0.04, 0.11]. 82% of the sample-wise differences were above zero.

The average difference in loss aversion was 0.084 (indicating more intense loss aversion), with a 95% HDI of [-0.03, 0.20], and 92% of sample-wise differences above zero. This is somewhat stronger of an effect than that observed with the Identity effect in Model 1.

The average difference in choice consistency was 0.16 (95% HDI: [-0.03, 0.36]) with 95% of sample-wise differences above zero.

**Model Comparison**. We assessed and compared the degree to which Models 1 and 2 fit the data. The average summed log likelihood of Model 1 across the 10,000 samples was -5683.3 (95% CI = [-5704, -5664]), while that of Model 2 was -5378.4 (95% CI = [-5409, -5350]; higher values [i.e. closer to zero] are better), establishing that Model 2 consistently fit the data better in an absolute sense. However, Model 2 was also much more complex than Model 1 (Model 1 had 186 parameters; Model 2 had 357 parameters). We therefore compared model fit using the expected log pointwise predictive density (ELPD) widely applicable information criterion (WAIC) (16), which can be understood as an approximate estimate of the pointwise out-of-sample prediction accuracy. The ELPD WAIC of the two models established that Model 2 performed better overall (Model 1 ELPD WAIC = -5796.2; Model 2 ELPD WAIC = -5559.5; higher values [i.e. closer to zero] are better; difference = -236.6, SE = 24.2).

We also evaluated the fit of a simple non-social control model. This model features no social context variables, only prospect theory parameters capturing risk attitudes, loss aversion, and choice consistency, with each specified in a random effects framework (i.e. with a group-level distribution which constrains individual-level estimates). The model fit well, with chains converging and R-hat values of 1. This model had 177 parameters (6 group-level parameters governing means & variances for the distributions of rho, lambda, and mu, and 3 x 57 parameters for each of the individual-level values of rho, lambda, and mu). The average summed log likelihood of this model (which we call Model 0) across the 10,000 samples was -5804.3 (95% CI = [-5824, -5786]), indicating a worse absolute fit, compared to Models 1 or 2. The ELPD WAIC of Model 0 was -5909.8, a difference of -350.2 (SE = 30.6) with Model 2, and -113.58 (SE = 18.3) with Model 1.

Figure S1.  Probability of risk-taking as a function of expected value difference in risky vs. safe options for Model 1. This plot depicts the predicted probability of making a risky choice as a function of the difference in expected value between the risky and safe options for each of the five conditions in which people made choices in this study (self, friend-only, friend-shared, stranger-only, stranger-shared).  Predicted probabilities were calculated using the mean group-level parameter values from Model 1 (see Figure 3 and Table 1, main manuscript). The effects captured by Model 1 and described in the manuscript are evident in the figure, including weak effects of sharing on any aspect of risk-taking (dashed and solid lines nearly overlap), and greater consistency for others (and especially for friends; red and blue lines are steeper than the black line). Note that this figure cannot accurately represent the nonlinearities captured by some of Model 1’s parameters, and necessarily superimposes multiple distinct effects into a single behavioral metric (p(Risky Choice)).

Table S1. Summary of social change effects on risk attitudes, loss aversion and choice consistency for Model 1. Values are implied mean estimates (with 95% HDI values in square brackets) generated by change terms when combined with the baseline estimates (see Results; note that HDIs include all sources of variability, including estimates of baseline values, change terms, etc.). Consistent effects, i.e. where the HDI does not include 0, are identified with cell-highlighting. Values of rho (*ρ*) capture risk attitudes; when *ρ* = 1, participants are risk-neutral. Values < 1 indicate risk aversion while values > 1 indicate risk-seeking (for gains; opposite pattern for losses). Values of lambda (λ) capture loss aversion. When *λ* = 1, participants are gain-loss neutral. Values > 1 indicate loss aversion, while those < 1 indicate gain-seeking. Values of mu (*μ*) capture consistency, with higher values indicating greater consistency.

|  | **Risk attitudes (ρ)** | **Loss aversion (λ)** | **Consistency (μ)** |
| --- | --- | --- | --- |
| Self | 1.07 [0.96, 1.18] | 1.78 [1.42, 2.19] | 18 [13.71, 22.07] |
| Other* | 0.96 [0.86, 1.06] | 1.90 [1.52, 2.34] | 28.3 [22.0, 34.7] |
| Sharing^ | Shared: 0.94 [0.85, 1.04]  Other only: 0.97 [0.88, 1.08] | Shared: 1.92 [1.51, 2.34]  Other only: 1.88 [1.49, 2.31] | Shared: 29.6 [23.0, 36.7]  Other only: 27.1 [21.0 33.3] |
| Identity^#^ | Friend: 1.03 [0.92 1.14]  Stranger: 0.92 [0.82, 1.03] | Friend: 1.85 [1.46, 2.27]  Stranger: 1.92 [1.50, 2.36] | Friend: 32.3 [24.4, 40.4]  Stranger: 22.7 [16.9 28.2] |

** ignoring effects of identity and sharing*

*^ ignoring effects of identity*

*^#^ assuming “other-only” for sharing*

**Supplemental References**

1. Sokol-Hessner P, Hsu M, Curley NG, Delgado MR, Camerer CF, Phelps EA. Thinking like a trader selectively reduces individuals’ loss aversion. Proceedings of the National Academy of Sciences of the United States of America. 2009 Mar;106(13):5035–40.

2. Sokol-Hessner P, Camerer CF, Phelps EA. Emotion regulation reduces loss aversion and decreases amygdala responses to losses. Social Cognitive and Affective Neuroscience. 2013 Mar;8(3):341–50.

3. Sokol-Hessner P, Hartley CA, Hamilton JR, Phelps EA. Interoceptive ability predicts aversion to losses. Cognition and Emotion. 2015 May 19;29(4):695–701.

4. Pachur T, Mata R, Hertwig R. Who Dares, Who Errs? Disentangling Cognitive and Motivational Roots of Age Differences in Decisions Under Risk. Psychological Science. 2017;28(4):504–18.

5. Rutledge RB, Skandali N, Dayan P, Dolan RJ. Dopaminergic Modulation of Decision Making and Subjective Well-Being. The Journal of Neuroscience. 2015;35(27):9811–22.

6. Booij A, van de Kuilen G. A parameter-free analysis of the utility of money for the general population under prospect theory. Journal of Economic Psychology. 2009;30(4):651–66.

7. Bates D, Maechler M, Walker S, Haubo Bojesen Christense R, Singmann H. Linear mixed-effects modles using Eigen and S4. 2014 Apr;1–90.

8. Kuznetsova A, Brockhoff PB, Haubo Bojesen Christense R. Tests for random and fixed effects for linear mixed effect models (lmer objects of lme 4 package). 2014 Feb;1–17.

9. Fareri DS, Niznikiewicz MA, Lee VK, Delgado MR. Social Network Modulation of Reward-Related Signals. Journal of Neuroscience. 2012 Jun;32(26):9045–52.

10. Fareri DS, Chang LJ, Delgado MR. Computational substrates of social value in interpersonal collaboration. Journal of Neuroscience. 2015 May;35(21):8170–80.

11. Brooks HR, Sokol-Hessner P. Quantifying the immediate computational effects of preceding outcomes on subsequent risky choices. Sci Rep. 2020 Dec;10(1):9878.

12. Nilsson H, Rieskamp J, Wagenmakers E-J. Hierarchical Bayesian parameter estimation for cumulative prospect theory. Journal of Mathematical Psychology. 2011 Feb 1;55(1):84–93.

13. Scheibehenne B, Pachur T. Using Bayesian hierarchical parameter estimation to assess the generalizability of cognitive models of choice. Psychon Bull Rev. 2015 Apr 1;22(2):391–407.

14. Sokol-Hessner P, Raio CM, Gottesman SP, Lackovic SF, Phelps EA. Acute stress does not affect risky monetary decision-making. Neurobiology of Stress. 2016 Dec;5:19–25.

15. Tversky A, Kahneman D. Advances in prospect theory: Cumulative representation of uncertainty. J Risk Uncertainty. 1992 Oct 1;5(4):297–323.

16. Vehtari A, Gelman A, Gabry J. Practical Bayesian model evaluation using leave-one-out cross-validation and WAIC. Stat Comput. 2017 Sep 1;27(5):1413–32.
